# Differences in wait-list mortality: Temporary vs durable circulatory support devices

**DOI:** 10.1016/j.jhlto.2025.100312

**Published:** 2025-06-02

**Authors:** Mahwash Kassi, Salma Zook, Duc Nguyen, Katelyn Ingram, Sapna Legha, Rayan Yousefzai, Ju Kim, Imad Hussain, Cindy M. Martin, Janardhana Gorthi, Adeel Ahsan Syed, Nadia Fida, Arvind Bhimaraj, Edward A. Graviss, Ashrith Guha

**Affiliations:** a Department of Cardiology, Houston Methodist Hospital – DeBakey Heart and Vascular Center, Houston, TX; b Department of Pediatrics, Baylor College of Medicine, Houston, TX; c Department of Pathology and Genomic Medicine, Houston Methodist Research Institute, Houston, TX; d Department of Surgery, Houston Methodist Hospital, Houston, TX

**Keywords:** Heart transplantation, Mechanical circulatory support, UNOS allocation system, Waitlist mortality, Propensity score-matching

## Abstract

**Background:**

In 2018, changes in the United Network for Organ Sharing (UNOS) allocation system led to a shift in practices, making durable left ventricular assist devices less desirable as a bridge to transplantation compared to temporary mechanical circulatory support. This study compares the composite outcome of waitlist mortality and delisting incidence at 1 year between these two support types.

**Methods:**

All actively listed adult patients on mechanical circulatory support listed for heart transplantation under the current UNOS system from October 2018 to October 2021 were included, excluding those with right ventricular devices, biventricular devices, total artificial hearts, and extracorporeal membrane oxygenators. The primary outcome was the composite of waitlist mortality and delisting due to clinical deterioration at 1 year. Survival analysis was conducted using Kaplan-Meier curves and multivariable Cox regression.

**Results:**

A total of 4,569 patients were included, with 1,877 on temporary mechanical circulatory support and 2,692 on left ventricular assist devices. Propensity-score matching was performed on 660 patients divided into two groups. The event rate was lower in the left ventricular assist device group compared to the temporary mechanical circulatory support group (15.9% vs 35.2%, *p* < 0.001). Temporary mechanical circulatory support had a significantly higher multivariable hazard ratio (HR) for outcome events (HR 3.37, *p* < 0.001). The HeartMate 3 (HM3) had the best outcomes compared to all other device types.

**Conclusion:**

In this propensity-score-matched analysis, durable mechanical circulatory support had better outcomes than temporary mechanical circulatory support. HM3 had the lowest risk of composite outcomes.

## Background

Advancements in mechanical circulatory support (MCS) devices have revolutionized the field of organ transplantation. Patients may be bridged to transplantation with either temporary MCS (tMCS) devices, such as intra-aortic balloon pumps (IABPs) and Impellas, or durable MCS devices (e.g., left ventricular assist devices, LVADs).^1^ These devices provide critical support to patients with end-stage heart failure while they await a suitable donor organ.^2^ Since the United Network for Organ Sharing (UNOS) revised the Uniform Anatomical Gift Act in 2006, overall survival for patients on the transplant waitlist has significantly improved. However, waitlist mortality remains substantial, ranging from 5% to 39%, some of which may be attributed to device-related complications.^3^ Complications attributed to temporary vs durable MCS may vary depending on device type and duration of support. Therefore, it is crucial to identify and understand differences in waitlist mortality between patients bridged with temporary and durable MCS devices.

Over several decades, durable MCS, particularly LVADs, have increasingly been used to bridge patients to transplantation as the devices became better and less prone to complications. For example, in the MOMENTUM trial, the HeartMate 3 (HM3) LVAD showed the best survival rates and the lowest adverse event profile.^4^, ^5^ It was anticipated that the results of this study would increase enthusiasm for the adoption of LVAD technology for patients with end-stage heart failure. However, the years following the implementation of the new allocation system changed this trajectory, and instead, the overall rate of LVAD implantation has been drastically reduced.^6^

The new allocation policy (implemented in 2018) resulted in more transplants and lower wait times to transplant without jeopardizing post-transplant survival.^7^, ^8^ The goal of these changes was also to reduce wait-list mortality by ensuring that the most critical patients receive priority for heart transplantation.^9^ Patients with imminent risk of death from cardiogenic shock are often stabilized with tMCS devices. Recognizing this risk, tMCS patients were prioritized in the current allocation system. Those with durable MCS are considered more stable; therefore, they are listed lower on the UNOS allocation system unless there is a complication.^10^ One of the consequences of this particular change regarding device type resulted in an increase in the use of tMCS devices, particularly IABP, from 8% to 26%,^11^ and a decrease in durable LVADs from 45% to 32%.^10^

Several studies seem to suggest that these policy changes led to changes in clinical practice—a response to the revised allocation system.^12^ This could be a form of “practice bias,” whereby tMCS is selected over durable MCS as a bridge to transplant by sheer nature of the transplant priority rather than patient need.^10^, ^13^ The post-transplant outcomes in patients bridged with tMCS and LVAD are similar,^2^ but a given patient’s wait-list survival is equally important. Currently, there is no direct comparison of waitlist mortality between tMCS and durable LVAD approaches since the new allocation system was implemented. This information is critical to directing the care of patients who await transplantation. Here, we sought to compare the composite outcomes of wait-list mortality and risk of delisting due to clinical deterioration between patients bridged with either tMCS or durable LVAD in a propensity score-matched analysis. In an additional sub-analysis, we compared HM3 LVAD with all other devices for these composite outcomes.

## Materials and methods

### Study population

This study strictly adheres to the ISHLT Ethics Statement and does not involve any transplants performed in violation of these guidelines. A query was conducted for patients on MCS between October 2018 and October 2021 in the UNOS database. This includes patients who were listed before October 2018 and remained on the waitlist during this specified period and included new patients who were added to the waitlist. The inclusion criteria were diagnosis of heart failure and receipt of temporary (Impella and IABP) or durable (HM3, HM II, and HeartWare Ventricular Assist Device [HVAD]) MCS as a bridge to transplant. Patients supported with right ventricular assist devices, biventricular assist devices, and total artificial hearts were excluded. Even though an extracorporeal membrane oxygenator (ECMO) is considered a form of tMCS, patients who received this support were excluded from our analysis because ECMO is traditionally used for biventricular support and in patients with respiratory failure as well as cardiogenic shock. In addition, it is known that outcomes are worse for patients on ECMO.^14^, ^15^ From this query, 4,569 patients were identified for a retrospective, cross-sectional study (Supplementary Figure 1). Patients were followed up until death, transplant, or removal from the list due to clinical deterioration. A summary table of patients who underwent a status change from the time of listing to the time of delisting or death is provided in the supplement (Supplementary Table 1). The institutional board review of XXXX approved this Health Insurance Portability and Accountability Act-compliant study. The primary outcome for this analysis was a composite outcome of waitlist mortality and delisting due to a deteriorating medical condition within 1 year of listing.

### Statistical analysis

Participants were categorized into two primary groups: tMCS and durable MCS. For detailed sub-analysis, these patients were further subdivided into five device-specific groups: IABP, Impella, HVAD, HM II, and HM 3. Descriptive statistics, including frequencies and proportions, were employed to present demographic and clinical characteristics.

The comparisons between groups were performed using chi-square or Fisher’s exact tests for categorical variables and the Wilcoxon rank-sum test for continuous variables, as deemed suitable. Kaplan-Meier curves were generated to illustrate the cumulative incidence of the composite outcome within one year of listing.

Cox regression modeling was used to identify potential characteristics associated with the composite outcome (defined as pre-transplant mortality or removal due to a deteriorated medical condition) within 1 year of listing. Variables for multivariable models were selected based on their clinical importance, supplemented by the least absolute shrinkage and selection operator method with cross-validation selection option. The discriminatory power of the model was determined by C-statistics.

This analytical procedure was reiterated for several subgroups, specifically comparing each of the following subgroups to HM3: tMCS, HM II, HVAD, Impella, and IABP. All statistical analyses were executed using Stata version 17.0 (StataCorp LLC, College Station, TX, USA), with a *p*-value of < 0.05 considered statistically significant.

### Propensity score estimation

Propensity score matching (PSM) was implemented to control for potential confounding variables and to establish the comparability of the temporary and durable MCS groups. PSM was based on the covariates from the UNOS database at the time of listing, shown in Supplementary Table 2. After PSM, the balance of the covariates was assessed using the percent standardized bias. As delineated in Supplementary Table 2, the analysis demonstrated a satisfactory balance between the tMCS and durable MCS groups, ensuring their comparability. The final propensity score-matched cohort consisted of 660 patients, equally distributed between the tMCS group and the durable MCS group. Finally, a sensitivity analysis was conducted using PSM to compare HM3 with other device types, incorporating the same covariates used at listing.

## Results

### Study population and patient characteristics

A total of 4,569 patients active on the transplant list between 2018 and 2021 satisfied the inclusion criteria (Supplementary Figure 1). Among these patients, 1,877 (41.1%) were supported by tMCS, while 2,692 (58.9%) received durable MCS. Supplementary Table 2 demonstrates the differences between patients in the tMCS and durable MCS groups. In general, the tMCS patients tended to be older [57 (47-64) years vs 56 (45-62) years, *p* < 0.001], had lower Body Mass Index (BMI) [26.7 (23.5-30.4)% vs 29.9 (26.3-33.4) %, *p* < 0.001]. The proportion of female patients was slightly higher in the tMCS group (24%) than in the durable MCS group (20.7%). African American patients represented a higher proportion in the durable MCS group than the tMCS group (31.5% vs 27.8%), *p* < 0.001.

### Comparison of event rates in the unmatched cohort

**Figure 1:**
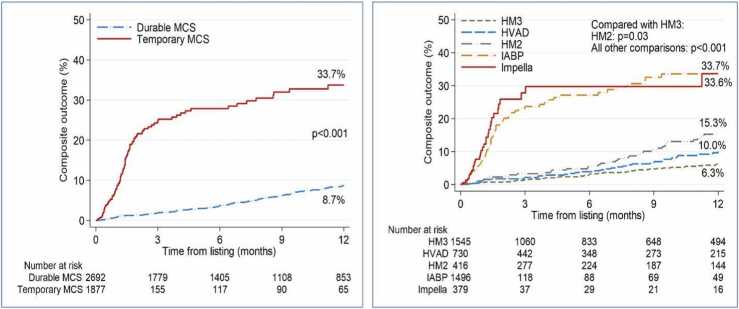
One-year Kaplan Meier curves of composite event incidence after listing. Left: Comparison between tMCS (33.7%) and Durable MCS (8.7%) groups. *p* < 0.001. Right: Device-specific event rates: HM3 (6.3%), tMCS-IABP (33.7%), Impella (33.6%), HM2 (15.3%), and HVAD (10%). HM2, HeartMate 2; HVAD, HeartWare Ventricular Assist Device.

In the unmatched cohort, the distribution of outcomes between the two groups is shown in Table 1. The tMCS group had a higher composite outcome than the durable MCS group within the first year of listing (6.7% vs 4.8%, *p* < 0.001). As illustrated in Figure 1, the Kaplan-Meier curve shows that event-free survival was significantly better in the durable MCS group (8.7%) compared to the tMCS group (33.7%), *p* < 0.001.

**Table 1:** Comparison of Event Rates Between the Temporary MCS and LVAD Groups in the Pre-Matched Cohort

Outcomes	Total (*N* = 4569)	Durable MCS (*n* = 2692)	Temporary MCS (*n* = 1877)	*p*-value
Any pre-transplant death	210 (4.6)	131 (4.9)	79 (4.2)	**0.30**
Days to death (in patient who died), median (IQR)	168.5 (37.0, 439.0)	335.0 (152.0, 568.0)	40.0 (16.0, 70.0)	**<0.001**
Pre-transplant death within 1 year from listing	148 (3.2)	71 (2.6)	77 (4.1)	**0.01**
Any death while on waiting list	120 (2.6)	71 (2.6)	49 (2.6)	**0.96**
Days to death while on waiting list, median (IQR)	113.5 (29.0, 339.0)	247.0 (99.0, 568.0)	33.0 (13.0, 53.0)	**<0.001**
Any death while on waiting list within 1 year	92 (2.0)	44 (1.6)	48 (2.6)	**0.03**
Any removal due to condition deteriorated	197 (4.3)	120 (4.5)	77 (4.1)	**0.56**
Days from listing to removal, median (IQR)	128.0 (31.0, 330.0)	255.5 (123.5, 428.5)	29.0 (12.0, 46.0)	**<0.001**
Removal due to condition deteriorated within 1 year from listing	154 (3.4)	81 (3.0)	73 (3.9)	**0.10**
Any composite outcome (pre-transplant death or removal due to condition deteriorated	347 (7.6)	217 (8.1)	130 (6.9)	**0.15**
Days to composite outcome (in patient who had event), median (IQR)	147.0 (32.0, 387.0)	287.0 (136.0, 543.0)	32.0 (12.0, 53.0)	**<0.001**
Composite outcome within 1 year from listing	254 (5.6)	129 (4.8)	125 (6.7)	**0.01**

Abbreviation: IQR, Interquartile range.

Upon comparing event rates among different device types to HM3 within the pre-matched cohort, some clear patterns emerged. The composite outcome at 1-year was notably higher for the tMCS group compared to the HM3 group, at 6.7% (125 cases) vs 3.6% (55 cases), respectively (*p* < 0.001). A similar trend was observed when comparing HM3 with other durable devices (i.e., HM II or HVAD). In particular, the composite outcome in the group of patients with either HM II or HVAD was significantly higher than in the HM3 group (6.5% (74 cases) vs 3.6% (55 cases), *p* < 0.001). The Kaplan-Meier survival curves in this pre-matched analysis show that HM3 consistently had better survival than all other device types (Figure 1).

Results from univariable Cox regression analysis for the composite outcome at 1 year showed that patients with tMCS had a 6.94-fold greater chance of experiencing the event than patients with durable MCS (HR = 6.94, 95% CI: 5.36-8.98, *p* < 0.001). Patients with tMCS had about 9.06 times greater risk of experiencing the event than those with HM3 (HR = 9.06, 95% CI: 6.52-12.60, *p* < 0.001). Comparing patients with HVAD/HM II to those with HM3, the risk of experiencing the event was approximately 1.92 times greater (HR = 1.92, [95% CI: 1.36-2.73], *p* < 0.001) in the HVAD/HM ll group.

In our multivariable analysis comparing tMCS and durable MCS (*n* = 4,418), we found several key factors significantly associated with the occurrence of composite events shown in Table 2 Model 1. Patients who received tMCS were significantly more at risk than those who received durable MCS, with an adjusted HR of 8.64 (95% CI: 5.14-14.53, *p* < 0.001).

**Table 2:** Characteristics Associated With Composite Outcome Event Within 1 Year From Listing, Multivariable Cox Regression (Pre-Matched Cohorts)

	Multivariable, model 1 (*n* = 4418)		Multivariable, model 2 (*n* = 4418)	
	HR (95% CI)	*p*-value	HR (95% CI)	*p*-value
*Temporary MCS vs Durable MCS*			--	--
Durable MCS	(Reference)		--	--
Temporary MCS	8.64 (5.14, 14.53)	**<0.001**	--	--
*Device group*	--	--		
HM3	--	--	(reference)	
HVAD/HM2	--	--	4.47 (2.70, 7.40)	**<0.001**
IABP	--	--	13.57 (6.95, 26.50)	**<0.001**
Impella	--	--	19.35 (8.99, 41.67)	**<0.001**
Age at listing (years)	1.02 (1.01, 1.03)	**0.003**	1.02 (1.01, 1.03)	**0.001**
BMI at listing (kg/m^2^)	0.99 (0.96, 1.02)	**0.47**	0.99 (0.96, 1.01)	**0.33**
Non-O blood group	1.18 (0.91, 1.52)	**0.21**	1.19 (0.92, 1.53)	**0.19**
Restrictive cardiomyopathy	1.55 (0.79, 3.05)	**0.20**	1.59 (0.81, 3.12)	**0.18**
Diabetes	1.10 (0.84, 1.44)	**0.48**	1.10 (0.84, 1.44)	**0.48**
Adult status 1-3 at listing	2.90 (2.09, 4.01)	**<0.001**	2.75 (2.00, 3.79)	**<0.001**
Most recent creatinine on listing (mg/dL)	1.27 (1.17, 1.39)	**<0.001**	1.28 (1.17, 1.40)	**<0.001**
Mean PA pressure at listing (mmHg)	1.02 (1.01, 1.03)	**0.01**	1.02 (1.01, 1.03)	**0.004**
Ventilation at listing	4.64 (2.41, 8.91)	**<0.001**	4.78 (2.47, 9.26)	**<0.001**
	**C-statistic = 0.81**		**C-statistic = 0.81**	

Abbreviations: BMI, Body Mass Index; HM2, HeartMate 2; HM3, HeartMate 3; HR, hazard ratio; HVAD, HeartWare Ventricular Assist Device; IABP, intra-aortic balloon pumps; MCS, Mechanicalcirculatory support devices.

Table 2 Model 2 demonstrates predictors significantly associated with the occurrence of composite events when comparing HM3 with other durable MCS device types in a multivariable analysis. In comparison to patients using the reference HM3 device, patients with HVAD/HM II had a 4.47 times higher hazard ratio (HR) (95% CI: 2.70-7.40, *p* < 0.001). For those who used IABP and Impella devices, the risk was even higher, with HRs of 13.57 (95% CI: 6.95-26.50, *p* < 0.001) and 19.35 (95% CI: 8.99-41.67, *p* < 0.001), respectively.

#### Propensity score-matched analyses

**Figure 2:**
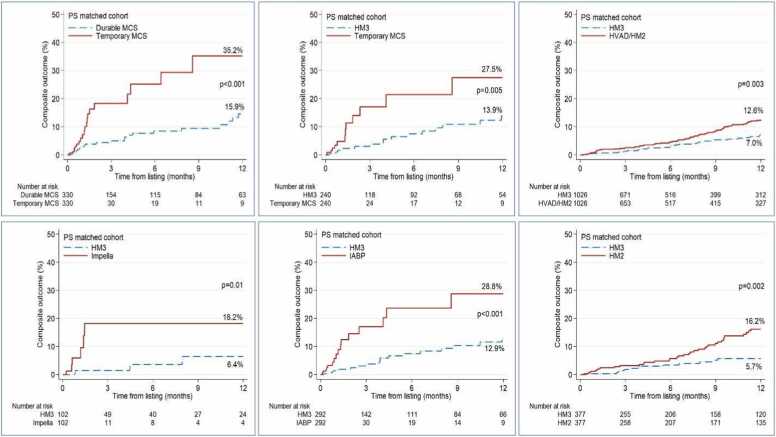
Kaplan Meier curves for the composite event incidence within 1 year from listing in the propensity-matched cohort.

In the propensity score-matched analysis, patients' characteristics are shown in Supplementary Table 3. The Kaplan-Meier plots for the composite outcome within one year of listing revealed a significantly higher incidence of composite events in the tMCS group (35.2%) compared to the durable MCS group (15.9%), *p* < 0.001 (Figure 2). The sensitivity analysis showed a lower composite outcome for HM3 as compared to tMCS, HVAD/HM II LVAD, HVAD, and HM II, highlighting the better clinical durability of the HM3 device.

Although our endpoint was limited to 1 year, we further explored the composite outcome for 3 years to understand the time frame where the risk of delisting or death would be higher in patients with durable LVAD compared to tMCS (Supplementary Figure 2). Here again, tMCS had a higher risk of the composite outcome when compared to durable MCS at any given point in time over 3 years.

#### HRs in the propensity score-matched cohort

We conducted multivariable Cox regression analyses comparing tMCS with durable MCS and subsequently, each MCS device with HM3 in a propensity score-matched cohort (Table 3). The aim was to predict the composite outcome event within one year of listing.

**Table 3:** Characteristics Associated With Composite Outcome Event Within 1 Year From Listing, Multivariable Cox Regression (PS Matched Cohorts)

	Temporary MCS vs Durable MCS Multivariable, model (*n* = 660)	
	HR (95% CI)	*p*-value
*Temporary MCS vs Durable MCS*		
Durable MCS	(Reference)	
Temporary MCS	3.37 (1.92, 5.94)	**<0.001**
BMI at listing (kg/m^2^)	0.98 (0.96, 1.00)	**0.07**
Adult status 1-3 at listing	3.43 (2.25, 5.24)	**<0.001**
Most recent creatinine on listing (mg/dL)	1.43 (1.21, 1.69)	**<0.001**
Ventilation at listing	4.93 (2.66, 9.13)	**<0.001**
	**C-statistic = 0.72**	

Abbreviations: BMI, Body Mass Index; HR, hazard ratio; MCS, Mechanical circulatory supportdevices.

In the first model (tMCS vs Durable MCS, *n* = 660), tMCS had a significantly higher hazard ratio (HR) of 3.37 (95% CI: 1.92-5.94, *p* < 0.001). Other notable findings included the slightly lower HR associated with a unit increase in BMI at listing (although this was not statistically significant), the significantly higher HR with Adult status 1-3 at listing, increased creatinine level, and ventilation at listing. The C-statistics for this model were 0.72, indicating acceptable model performance.

In subsequent models, all MCS devices had significantly higher HRs than the HM3, suggesting a greater risk of the composite outcome event within 1 year of listing. Increased creatinine levels and ventilation at listing generally led to higher HRs. In contrast, increased BMI at listing usually correlated with lower HRs, although not always statistically significant. Inotrope support at listing notably increased the HR in the third model. The C-statistics for these models ranged from 0.69 to 0.80 (Table 4), indicative of acceptable to good model performance.

**Table 4:** Comparison Between HM3 vs Other Device Types (PS Matched Cohort)

Comparison Groups	Sample Size (*n*)	Hazard Ratio (95% CI)	*p*-value	C-statistics
Temporary MCS vs Durable MCS	660	3.37 (1.92, 5.94)	**<0.001**	**0.72**
Temporary MCS vs HM3	480	2.95 (2.40, 3.63)	**<0.001**	**0.72**
HM2/HVAD vs HM3	2,052	1.93 (1.51, 2.47)	**<0.001**	**0.71**
Impella vs HM3	204	3.28 (1.56, 6.91)	**0.002**	**0.80**
IABP vs HM3	584	5.32 (3.10, 9.14)	**<0.001**	**0.73**
HM2 vs HM3	754	3.01 (1.83, 4.94)	**<0.001**	**0.74**
HVAD vs HM3	1,289	1.57 (1.05, 2.35)	**0.03**	**0.69**

BMI at listing (kg/m^2^): Influence varied across models; most notable in the Impella vs HM3 group (0.93 [0.87, 0.98], *p* = 0.01). Adult status 1-3 at listing: Significantly increased risk in all models; highest in HM2 vs HM3 group (3.97 [3.58, 4.40], *p* < 0.001). Most recent creatinine on listing (mg/dL): Increased risk across all models; most notable in HM2 vs HM3 group (1.54 [1.35, 1.75], *p* < 0.001). Ventilation at listing: Significantly increased risk in multiple groups; most notable in the HM2/HVAD vs HM3 group (86.92 [32.96, 229.23], *p* < 0.001).

Abbreviations: HM2, HeartMate 2; HM3, HeartMate 3; HVAD, HeartWare Ventricular Assist Device; IABP, intra-aortic balloon pumps; MCS, Mechanical circulatory support devices.

These findings highlight the clinical challenges associated with tMCS and devices such as HM II and HVAD. The significantly higher HRs observed with tMCS underscore the importance of careful patient selection and timely transition to more durable support when feasible. Furthermore, the superior outcomes associated with HM3 reinforce its role as the preferred durable MCS device in this population. These results emphasize the need for individualized decision-making in selecting MCS strategies to optimize waitlist survival and minimize the risk of adverse outcomes.

## Discussion

The use of tMCS devices has increased following changes in organ allocation policies, with comparable outcomes after heart transplants.^9^, ^15^ Our study sought to elucidate the disparities between tMCS and durable MCS to a composite outcome encompassing waitlist mortality and delisting due to worsening clinical status at the 1 year mark in the current era. Our pivotal findings include: (1) tMCS carries the highest risk of adverse composite outcomes of waitlist mortality and risk of delisting due to clinical deterioration, and (2) the HM3 device has the lowest risk of adverse events when compared to other device types.

Pre-transplant and post-transplant survival are equally important to any patient in need of a heart transplant. Even after changes to the allocation policies granted priority to tMCS recipients, the mortality rate on the waitlist and the risk of clinical decline remains notably high, particularly when juxtaposed with the HM3 durable LVAD device. This elevated risk might be attributed to potential complications tied to tMCS, such as stroke and limb ischemia.^16^ Increasingly, centers are adopting tMCS bridging approaches to expedite the transplantation process.^6^ However, such approaches should be employed judiciously. Conversely, considering the low event rate of the HM3, a bridge-to-transplant with durable MCS may be worthwhile if patients remain dependent on tMCS after a given time frame. However, the appropriate time frame to make that decision may be a matter of debate and may need a personalized patient approach. In addition, it is prudent to understand that a “double-bridged” approach may have its challenges. For some patients, it may take several years before they can be transplanted. In the current allocation system, LVAD patients are given a higher priority when they have suffered a complication. While tMCS portends a higher risk at any given time point when compared to durable MCS, our analysis showed that there is an incremental risk to patients on LVAD after being on support for 18 months. Therefore, newer iterations of heart allocation could include waitlist time on durable MCS as a modifiable factor to allow for a higher listing status around this time before a complication occurs. Not surprisingly, the risk is also high within the first year, most likely related to surgical complications such as stroke, RV failure, and renal failure. According to one study, the average time patients with LVAD wait until progress to a heart transplant is about fourteen months.^17^

The overall trend in reduction in LVAD implantation may have unintended outcomes, for example, a young patient who gets an LVAD first and then a heart transplant may have a longer survival period than if they were to get a transplant alone. This in turn will lead to a reduction in the number of additional years a stable transplant candidate could live on LVAD support before going for a heart transplant. Furthermore, certain patients with bridge-to-transplant implants may be eligible for recovery and explanation, which could postpone or eliminate the need for a heart transplant altogether.^18^

Regarding durable LVAD, our data suggest that the HM3 is the superior device. In the pre-allocation era, some studies suggested higher waitlist mortality and risk of clinical deterioration in patients with continuous-flow LVAD.^19^ In one propensity score-matched study of status two patients with or without LVAD, CF-LVAD patients had significantly higher waitlist mortality (14.6% vs 2.9%, *p* < 0.0001) and removal from the waitlist (20.9% vs 15.5%, *p* = 0.05).^19^ The reason for removal was clinical deterioration in 51.7% of the patients with LVAD and 24.7% in the no-LVAD group. These prior cohorts were predominantly comprised of HVAD and HM II. Our study findings are in line with prior published data, where HMII and HVAD are associated with more hemocompatibility adverse events, resulting in a higher risk of waitlist mortality and removal. HM II and HVAD are associated with higher rates of device-related complications, including pump thrombosis, bleeding, and infections, which contribute to increased morbidity and mortality. For instance, pump thrombosis rates in these devices have been reported to be significantly higher than in the HM3, often leading to the need for device replacement or urgent transplant. These complications not only increase the risk of waitlist mortality but also contribute to the higher likelihood of removal from the waitlist due to clinical deterioration. Despite the superiority of HM3 over other durable devices and known issues with other device types, particularly HVAD, the type of LVAD does not change the overall priority in the current listing system. Our analysis reflects that future iterations of the allocation system should allow for listing status to be commensurate with the durable MCS device type.

Our study has several limitations related to being a retrospective database analysis. The implementation of the allocation system is rather recent, and we have data limited to 2021. We anticipate the trends reported in this paper will change over time. In the current system, there is a growing use of exception criteria, which we cannot fully account for in the current dataset. Despite our efforts to balance observed confounders through matching, there may still be unobserved confounding variables affecting the results. We did, however, perform sensitivity analyses to assess the robustness of our findings to potential unobserved confounding.

## Financial support

This research received no specific grant from any funding agency in the public, commercial, or not-for-profit sectors. All authors have no conflict of interest or financial relationships to disclose.

## Data Sharing Statement

Data generated or analyzed during the study are available from the corresponding author by request.

## Declaration of Generative AI and AI-Assisted Technologies in the Writing Process

During the preparation of this work, the author(s) used [CHATGPT] to clean up language. After using this tool/service, the author(s) reviewed and edited the content as needed and take(s) full responsibility for the content of the publication.

## Declaration of Competing Interest

The authors declare that they have no known competing financial interests or personal relationships that could have appeared to influence the work reported in this paper.

## Footnotes

Appendix A Supplementary data associated with this article can be found in the online version at doi:10.1016/j.jhlto.2025.100312.
