# Supplementary material for: Differences in wait-list mortality: Temporary vs durable circulatory support devices
Source: JHLT Open. 2025 Jun 2;9:100312. doi: 10.1016/j.jhlto.2025.100312 (PMC12221459; doi:10.1016/j.jhlto.2025.100312)
Supplement: Supplementary file 1 — Supplementary material [file mmc1.docx]

**Differences in Wait-list Mortality: Temporary vs Durable Circulatory Support Devices**

Mahwash Kassi ^1^, MD, Salma Zook ^1^, MD, Duc Nguyen^2^, MD, PhD, Sapna Legha^1^, MD, Rayan Yousafzai^1^, MD, Ju Kim^1^, MD, Imad Hussain^1^, MD, Cindy M. Martin^1^, MD, Janardhana Gorthi^1^, MD, Adeel Ahsan Syed^1^, MD, Nadia Fida^1^, MD, Arvind Bhimaraj^1^, MD, Edward A. Graviss^3,4^, PhD, Ashrith Guha^1^, MD.

**Supplementary Materials:**

**Supplementary Figures and Figure Legends**

**
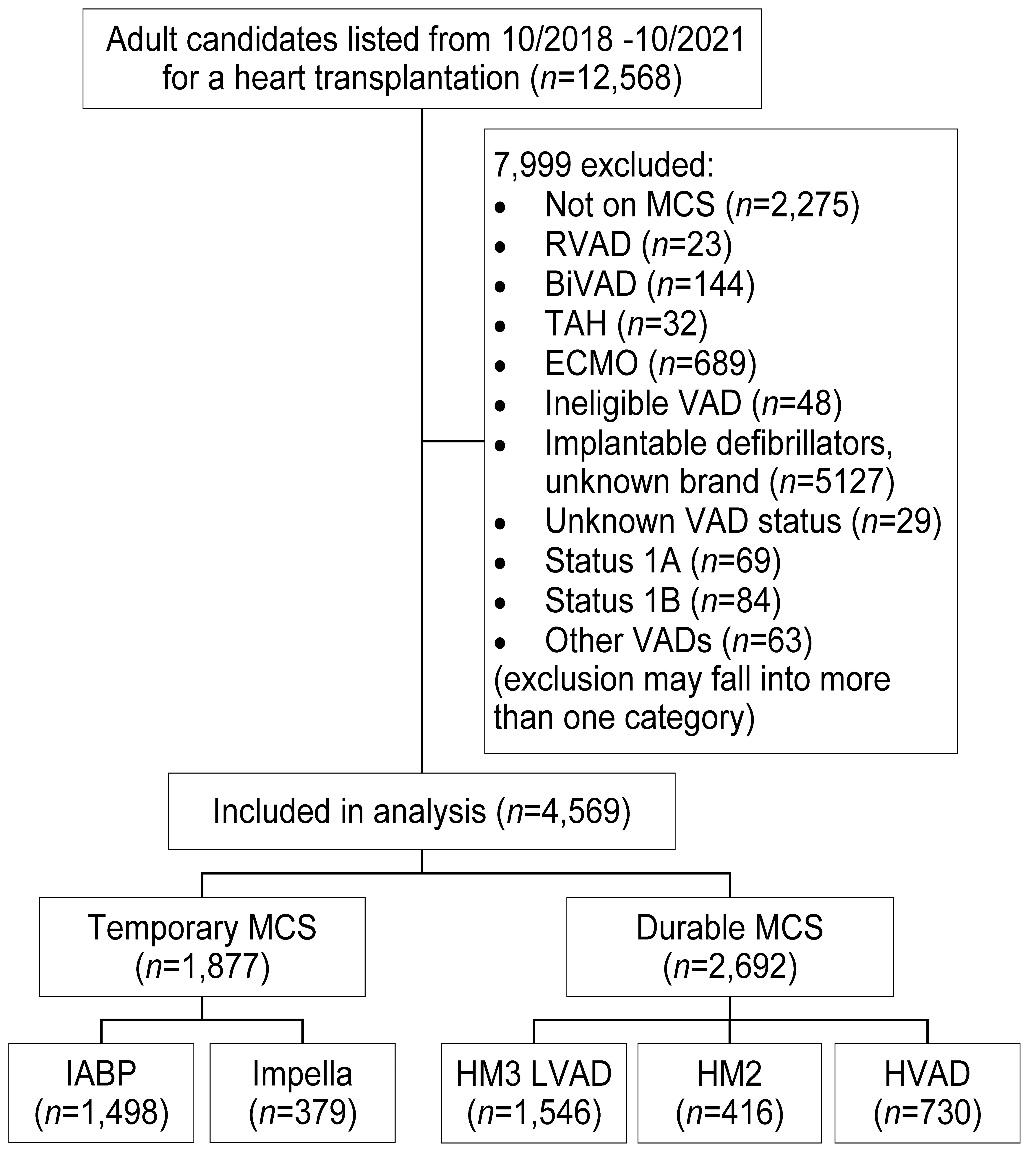
**

**Supplementary Figure 1. Flowchart of the study population**

**MCS: Mechanical circulatory support, HM3: HeartMate III,  LVAD: left ventricular assist device,  RVAD: right ventricular assist device,  BiVAD = LVAD + RVAD, TAH: total artificial heart, ECMO: extracorporeal membrane oxygenation**

**
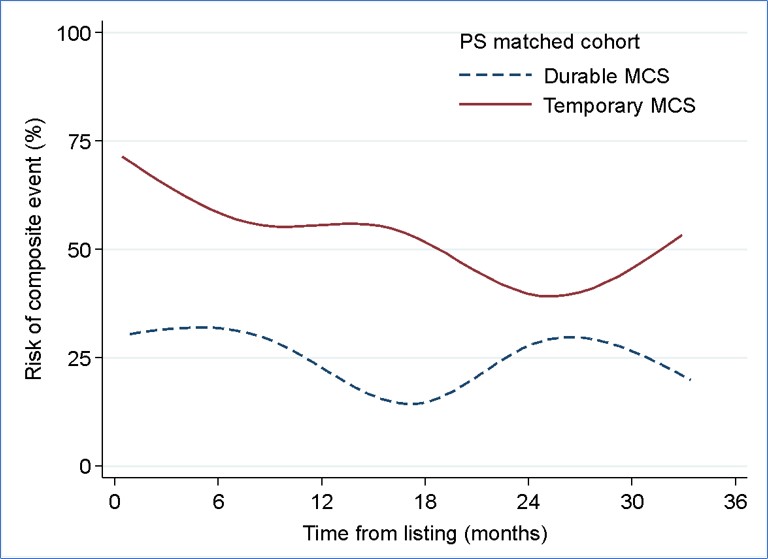
**

**Supplementary Figure 2**. Cubic spline plot for the risk of composite event within 36 months from listing, stratified by durable MCS vs temporary MCS.

**Supplementary Tables:**

**Supplementary Table 1**. Patients who underwent a status change from the time of listing to the time of delisting or death

| **Status at Listing** | **Status at delisting or death** | | | | | | | | |
| --- | --- | --- | --- | --- | --- | --- | --- | --- | --- |
|  | **Status 1A** | **Adult Status 1** | **Adult Status 2** | **Adult Status 3** | **Adult Status 4** | **Adult Status 5** | **Adult Status 6** | **Temporary Inactive** | **Total** |
| **Status 2** | 0 | 0 | 2 | 0 | 1 | 0 | 1 | 0 | 4 |
| **Adult status 1** | 0 | 35 | 3 | 0 | 0 | 0 | 0 | 3 | 41 |
| **Adult status 2** | 1 | 58 | 1,450 | 19 | 40 | 1 | 2 | 134 | 1,705 |
| **Adult status 3** | 0 | 16 | 123 | 339 | 87 | 0 | 1 | 70 | 636 |
| **Adult status 4** | 0 | 38 | 228 | 399 | 978 | 0 | 15 | 307 | 1,965 |
| **Adult status 5** | 0 | 1 | 3 | 0 | 1 | 0 | 0 | 0 | 5 |
| **Adult status 6** | 0 | 4 | 75 | 12 | 23 | 0 | 5 | 36 | 155 |
| **Temporary Inactive** | 0 | 2 | 10 | 10 | 10 | 0 | 0 | 26 | 58 |
| **Total** | 1 | 154 | 1,894 | 779 | 1,140 | 1 | 24 | 576 | 4,569 |

**Supplementary Table 2.** Patient characteristics (pre-matched cohorts)

| **Recipient characteristics** | **Total (n=4569)** | **Durable MCS (n=2692)** | **Temporary MCS (n=1877)** | **P-value** |
| --- | --- | --- | --- | --- |
| **Age at listing (years), median (IQR)** | 56.0 (46.0, 63.0) | 55.0 (45.0, 62.0) | 57.0 (47.0, 64.0) | **<0.001** |
| **Gender** |  |  |  | **0.01** |
| Female | 1008 (22.1) | 558 (20.7) | 450 (24.0) |  |
| Male | 3561 (77.9) | 2134 (79.3) | 1427 (76.0) |  |
| **Race/Ethnicity** |  |  |  | **<0.001** |
| White | 2557 (56.0) | 1517 (56.4) | 1040 (55.4) |  |
| Black | 1371 (30.0) | 849 (31.5) | 522 (27.8) |  |
| Hispanic | 433 (9.5) | 226 (8.4) | 207 (11.0) |  |
| Asian | 161 (3.5) | 68 (2.5) | 93 (5.0) |  |
| Other | 47 (1.0) | 32 (1.2) | 15 (0.8) |  |
|  |  |  |  |  |
| **BMI at listing (kg/m2), median (IQR)** | 28.6 (24.9, 32.4) | 29.9 (26.3, 33.4) | 26.7 (23.5, 30.4) | **<0.001** |
| **BSA at listing (m2), median (IQR)** | 2.0 (1.9, 2.2) | 2.1 (1.9, 2.2) | 2.0 (1.8, 2.1) | **<0.001** |
| **ABO blood group** |  |  |  | **<0.001** |
| A | 1629 (35.7) | 940 (34.9) | 689 (36.7) |  |
| B | 671 (14.7) | 351 (13.0) | 320 (17.0) |  |
| AB | 174 (3.8) | 89 (3.3) | 85 (4.5) |  |
| O | 2095 (45.9) | 1312 (48.7) | 783 (41.7) |  |
| **Thoracic diagnosis at listing** |  |  |  |  |
| Cardiomyopathy, ischemic | 1329 (29.1) | 849 (31.5) | 480 (25.6) | **<0.001** |
| Cardiomyopathy, non-ischemic | 2864 (62.7) | 1697 (63.0) | 1167 (62.2) | **0.55** |
| Restrictive cardiomyopathy | 95 (2.1) | 23 (0.9) | 72 (3.8) | **<0.001** |
| Coronary artery disease | 36 (1.1) | 22 (1.4) | 14 (0.9) | **0.17** |
| Hypertrophic cardiomyopathy | 38 (0.8) | 8 (0.3) | 30 (1.6) | **<0.001** |
| Valvular heart Disease | 24 (0.5) | 14 (0.5) | 10 (0.5) | **1.00** |
| Congenital heart disease | 45 (1.0) | 16 (0.6) | 29 (1.5) | **0.001** |
| Smoking | 2159 (47.3) | 1424 (52.9) | 735 (39.2) | **<0.001** |
| Diabetes | 1454 (31.9) | 886 (33.0) | 568 (30.3) | **0.06** |
| Previous malignancy | 305 (9.4) | 168 (10.4) | 137 (8.4) | **0.047** |
| Days on waiting list, median (IQR) | 45.0 (10.0, 283.0) | 195.0 (49.0, 444.0) | 10.0 (5.0, 23.0) | **<0.001** |
| **Heart allocation status at listing** |  |  |  | **<0.001** |
| Adult status 1-3 | 2382 (52.1) | 718 (26.7) | 1664 (88.7) | **<0.001** |
| Adult status 1 | 41 (0.9) | 18 (0.7) | 23 (1.2) | **0.06** |
| Adult status 2 | 1705 (37.3) | 124 (4.6) | 1581 (84.2) | **<0.001** |
| Adult status 3 | 636 (13.9) | 576 (21.4) | 60 (3.2) | **<0.001** |
| Adult status 4 | 1965 (43.0) | 1881 (69.9) | 84 (4.5) | **<0.001** |
| Most recent creatinine on listing, mg/dL, median (IQR) | 1.2 (1.0, 1.5) | 1.2 (1.0, 1.4) | 1.2 (1.0, 1.6) | **0.01** |
| Most recent eGFR on listing (mL/min/1.73m2), median (IQR) | 68.5 (51.6, 88.9) | 69.5 (53.7, 88.9) | 66.9 (47.8, 89.0) | **<0.001** |
| Most recent eGFR  on listing <60 (mL/min/1.73m2) | 1712 (37.6) | 951 (35.4) | 761 (40.6) | **<0.001** |
| Mean PA pressure at listing (mmHg), median (IQR) | 26.0 (19.0, 34.0) | 21.7 (17.0, 28.0) | 33.0 (27.0, 40.0) | **<0.001** |
| PA pulse pressure at listing (mmHg) , median (IQR) | 20.0 (15.0, 25.0) | 18.0 (14.0, 23.0) | 23.0 (18.0, 29.0) | **<0.001** |
| Mean PCW at listing (mmHg), median (IQR) | 16.0 (10.0, 24.0) | 12.0 (8.0, 18.0) | 24.0 (18.0, 30.0) | **<0.001** |
| Cardiac output (L/min), median (IQR) | 4.4 (3.5, 5.2) | 4.7 (4.0, 5.4) | 3.8 (3.0, 4.7) | **<0.001** |
| cardiac power index at listing, median (IQR) | 0.1 (0.1, 0.2) | 0.1 (0.1, 0.1) | 0.1 (0.1, 0.2) | **<0.001** |
| TPG at listing, median (IQR) | 9.0 (6.0, 12.0) | 9.0 (7.0, 11.0) | 10.0 (6.0, 14.0) | **<0.001** |
| PVR at listing, median (IQR) | 2.1 (1.4, 3.0) | 1.9 (1.3, 2.6) | 2.6 (1.6, 3.9) | **<0.001** |
| DPG at listing, median (IQR) | 2.0 (-1.0, 4.0) | 2.0 (0.0, 4.0) | 1.0 (-2.0, 4.0) | **<0.001** |
| Inotrope at listing | 1286 (28.1) | 131 (4.9) | 1155 (61.5) | **<0.001** |
| Ventilation  at listing | 41 (0.9) | 8 (0.3) | 33 (1.8) | **<0.001** |

**Supplementary Table 3.** Patient characteristics (propensity score matched cohort)

| **Recipient characteristics** | **Total (** **(N=660))** | **Durable MCS (** **(n=330))** | **Temporary MCS (** **(n=330))** | **P-value** |
| --- | --- | --- | --- | --- |
| **Age at listing (years), median (IQR)** | 57.0 (48.0, 63.0) | 57.0 (48.0, 63.0) | 58.0 (48.0, 64.0) | 0.41 |
| **Gender** |  |  |  |  |
| Female | 143 (21.7) | 72 (21.8) | 71 (21.5) | 0.93 |
| Male | 517 (78.3) | 258 (78.2) | 259 (78.5) | 0.93 |
| **Race/Ethnicity** |  |  |  |  |
| White | 387 (58.6) | 191 (57.9) | 196 (59.4) | 0.69 |
| Black | 181 (27.4) | 92 (27.9) | 89 (27.0) | 0.79 |
| Hispanic | 61 (9.2) | 31 (9.4) | 30 (9.1) | 0.89 |
| Asian | 25 (3.8) | 14 (4.2) | 11 (3.3) | 0.54 |
| Other | 6 (0.9) | 2 (0.6) | 4 (1.2) | 0.41 |
| **BMI at listing (kg/m2), median (IQR)** | 28.7 (25.0, 32.6) | 28.5 (24.9, 32.5) | 28.9 (25.1, 32.7) | 0.24 |
| **BSA at listing (m2), median (IQR)** | 2.0 (1.9, 2.2) | 2.0 (1.9, 2.2) | 2.0 (1.9, 2.2) | 0.17 |
| **ABO blood group** |  |  |  |  |
| A | 229 (34.7) | 114 (34.5) | 115 (34.8) | 0.94 |
| B | 114 (17.3) | 58 (17.6) | 56 (17.0) | 0.94 |
| AB | 28 (4.2) | 11 (3.3) | 17 (5.2) | 0.84 |
| O | 289 (43.8) | 147 (44.5) | 142 (43.0) | 0.25 |
| **Thoracic diagnosis at listing** |  |  |  |  |
| Cardiomyopathy, ischemic | 223 (33.8) | 112 (33.9) | 111 (33.6) | 0.94 |
| Cardiomyopathy, non-ischemic | 374 (56.7) | 187 (56.7) | 187 (56.7) | 1.00 |
| Restrictive cardiomyopathy | 17 (2.6) | 9 (2.7) | 8 (2.4) | 0.81 |
| Coronary artery disease | 8 (1.5) | 4 (1.6) | 4 (1.4) | -- |
| Hypertrophic cardiomyopathy | 5 (0.8) | 2 (0.6) | 3 (0.9) | 0.65 |
| Valvular heart Disease | 4 (0.6) | 2 (0.6) | 2 (0.6) | 1.00 |
| Congenital heart disease | 6 (0.9) | 3 (0.9) | 3 (0.9) | 1.00 |
| Smoking | 313 (47.4) | 148 (44.8) | 165 (50.0) | 0.19 |
| Diabetes | 314 (47.6) | 154 (46.7) | 160 (48.5) | 0.64 |
| Previous malignancy | 238 (36.1) | 116 (35.2) | 122 (37.0) | 0.63 |
| Days on waiting list, median (IQR) | 56 (10.4) | 31 (12.4) | 25 (8.6) | -- |
| **Heart allocation status at listing** | 22.0 (8.0, 119.0) | 76.5 (14.0, 274.0) | 13.0 (6.0, 27.0) | -- |
| Adult status 1-3 | 8 (1.2) | 5 (1.5) | 3 (0.9) | 0.48 |
| Adult status 1 | 498 (75.5) | 252 (76.4) | 246 (74.5) | -- |
| Adult status 2 | 12 (1.8) | 10 (3.0) | 2 (0.6) | -- |
| Adult status 3 | 297 (45.0) | 59 (17.9) | 238 (72.1) | -- |
| Adult status 4 | 189 (28.6) | 183 (55.5) | 6 (1.8) | -- |
| Most recent creatinine on listing, mg/dL, median (IQR) | 107 (16.2) | 49 (14.8) | 58 (17.6) | -- |
| Most recent eGFR on listing (mL/min/1.73m2), median (IQR) | 1.2 (1.0, 1.5) | 1.2 (1.0, 1.6) | 1.2 (1.0, 1.5) | 0.89 |
| Most recent eGFR  on listing <60 (mL/min/1.73m2) | 65.3 (49.7, 87.5) | 63.6 (50.2, 87.8) | 65.9 (49.3, 87.2) | 0.70 |
| Mean PA pressure at listing (mmHg), median (IQR) | 271 (41.1) | 137 (41.5) | 134 (40.6) | -- |
| PA pulse pressure at listing (mmHg) , median (IQR) | 28.0 (22.0, 36.0) | 28.0 (22.0, 36.0) | 29.0 (22.0, 37.0) | 0.93 |
| Mean PCW at listing (mmHg), median (IQR) | 21.0 (16.0, 26.0) | 21.0 (17.0, 26.0) | 21.0 (16.0, 26.0) | 0.87 |
| Cardiac output (L/min), median (IQR) | 19.0 (13.0, 25.0) | 19.0 (12.0, 25.0) | 18.5 (14.0, 24.0) | 0.98 |
| cardiac power index at listing, median (IQR) | 4.2 (3.4, 5.1) | 4.2 (3.4, 5.1) | 4.1 (3.4, 5.0) | 0.90 |
| TPG at listing, median (IQR) | 0.1 (0.1, 0.2) | 0.1 (0.1, 0.2) | 0.1 (0.1, 0.2) | 0.65 |
| PVR at listing, median (IQR) | 10.0 (7.0, 13.0) | 10.0 (7.0, 12.0) | 10.0 (6.0, 13.0) | 0.84 |
| DPG at listing, median (IQR) | 2.3 (1.5, 3.3) | 2.3 (1.5, 3.2) | 2.3 (1.5, 3.4) | 0.85 |
| Inotrope at listing | 2.0 (-1.0, 4.0) | 2.0 (-1.0, 4.0) | 2.0 (-1.0, 5.0) | 0.40 |
| Ventilation  at listing | 157 (23.8) | 82 (24.8) | 75 (22.7) | 0.52 |
